# Surgical Outcomes and Predictors of Success in Esotropia: A Five-Year Retrospective Review From a Large UK Tertiary Centre

**DOI:** 10.7759/cureus.101907

**Published:** 2026-01-20

**Authors:** Shie Wei Chan, Chi Kit Yan, Hadi Masoodi, Salim Khan, Bhamy Hariprasad Shenoy

**Affiliations:** 1 Ophthalmology, Manchester Royal Eye Hospital, Manchester, GBR; 2 Medicine, Manchester University NHS Foundation Trust, Manchester, GBR; 3 Paediatric Ophthalmology, Manchester Royal Eye Hospital, Manchester, GBR

**Keywords:** esotropia, factors for success, ‘paediatric’, squint, squint correction surgery, strabismus surgery, success outcome

## Abstract

Purpose: To evaluate surgical outcomes and predictors of success in a mixed cohort of patients undergoing esotropia surgery at a large UK tertiary centre.

Methods: This retrospective chart review included all patients who underwent extraocular muscle surgery for esotropia between July 2020 and June 2025. Demographic data, esotropia subtype, visual acuity, preoperative deviation, surgical procedure, total surgical dose, and postoperative deviation at last follow-up were collected. The primary outcome was surgical success at six weeks, defined as postoperative deviation ≤10 prism dioptres (PD). Secondary outcomes included surgical dose response and factors associated with surgical success. Univariate and multivariate analyses were performed.

Results: Seventy patients met the inclusion criteria. Mean age at surgery was 8.0 ± 3.9 years, and mean preoperative deviation was 37.7 ± 13.4 PD. Bilateral medial rectus recession was the most common procedure (81.4%). Surgical success at six weeks was achieved in 50% of patients, with an overcorrection rate of 1.4%. In multivariate analysis, older age at surgery predicted a higher likelihood of success (OR 1.37, p = 0.014). The mean surgical dose response was 2.39 ± 1.95 PD/mm and was significantly higher among successful cases (p < 0.001). Larger preoperative deviation was independently associated with a higher dose response.

Conclusions: In this mixed-aetiology real-world cohort, early surgical success was adequate. Older age and higher surgical dose response were associated with better alignment outcomes, whereas sex, visual acuity, esotropia subtype, and procedure type were not predictive of success.

## Introduction

Esotropia is the most common form of childhood strabismus, with an estimated prevalence of 1-2% in the paediatric population [1]. It encompasses a heterogeneous group of disorders, including infantile, accommodative, partially accommodative, acquired non-accommodative, and sensory forms, each with differing aetiologies, clinical trajectories, and responses to treatment. Early misalignment can impair binocular development, reduce stereopsis, and contribute to amblyopia, underscoring the importance of timely diagnosis and appropriate management.

Infantile esotropia typically presents within the first six months of life and often requires early surgical intervention to optimise binocularity [2]. Partially accommodative esotropia (PAET) and acquired esotropias form a substantial proportion of referrals in later childhood and may require combined optical and surgical interventions. Despite surgery being the mainstay of treatment for persistent deviations, outcomes continue to vary widely between subtypes and across surgical approaches.

A number of cohort studies have reported favourable outcomes in more homogeneous patient populations. Surgical success rates of 50-90% have been reported in infantile esotropia, PAET, and acute acquired comitant esotropia (AACE) [2-5]. Dose-response characteristics in medial rectus recession surgery have also been shown to differ by aetiology, with higher responses reported in infantile ET and in augmented surgical dosing for AACE [6-9].

To address these gaps, we conducted a five-year retrospective review at a large UK tertiary paediatric ophthalmology centre. Our institution receives a broad range of esotropia phenotypes and surgical complexities, offering an opportunity to evaluate outcomes in a mixed, real-world cohort. Six-week alignment was chosen as the primary endpoint because it serves as a key predictor of long-term stability and is a pragmatic benchmark in clinical follow-up schedules. The primary objective of this study was to determine the rate of early surgical success in a heterogeneous, real-world cohort of esotropia patients treated at a tertiary referral centre. Secondary objectives were to identify preoperative and surgical predictors of successful alignment and to characterise the surgical dose-response relationship across different esotropia subtypes and surgical approaches. We hypothesised that predictors and dose-response patterns observed in homogeneous cohorts would be attenuated in this mixed population.

## Materials and methods

This retrospective chart review was conducted at the Manchester Royal Eye Hospital, a large tertiary paediatric ophthalmology centre, and included all patients who underwent extraocular muscle surgery for esotropia between July 2020 and June 2025. The project was undertaken as a registered clinical audit within the institution. Data collection was manually extracted from electronic patient records. Patients were eligible if they had a documented diagnosis of esotropia and had at least one postoperative alignment assessment at six weeks. Exclusion criteria included previous strabismus surgery, oblique muscle surgery, oblique muscle overaction, known neurological or neuromuscular disorders affecting ocular motility, craniofacial anomalies, or incomplete pre- or postoperative prism measurements. A total of 70 patients met the criteria and were included in the final analysis.

Demographic and clinical variables extracted included age at surgery, age at presentation, sex, esotropia subtype (accommodative, infantile, AACE, consecutive, sensory or cyclic), and visual acuity (VA) recorded using logMAR and classified using ICD-11 VA categories. Binocular status was not documented in the majority of patients. Extraocular motility limitations and refractive error were not collected. Preoperative distance deviation was recorded in prism dioptres (PD). Surgical data included procedure type: bilateral medial rectus recession (bMR), unilateral medial rectus recession with lateral rectus resection (R&R), unilateral medial rectus recession (uMR), unilateral lateral rectus resection (uLR) or bilateral medial rectus recession with unilateral lateral rectus resection (bMR & uLR), and the total surgical dose in millimetres. Post-operative complications were also recorded. Park’s surgical table was used as the primary nomogram for determining surgical dosage. Augmented dosing was not routinely performed, including large-angle deviations, AACE or high AC/C ratios. Minor intraoperative adjustments were occasionally made based on findings such as forced duction testing; however, these deviations were limited and surgeon-specific rather than systematic. Surgeries were performed by consultant paediatric ophthalmologists, senior fellows and trainees using a standard limbal approach or fornix incisions. Trainees and fellows performed under consultant supervision, with the surgical dosage decided by the consultant.

Postoperative alignment at six weeks was the primary assessment timepoint and was recorded in PD at distance fixation. The measurement was taken with the prism cover test and with correction at 6 m by clinicians. Surgical success was defined as a postoperative deviation of ≤10 PD, with any manifest exotropia classified as overcorrection. Secondary outcomes included the surgical dose response, calculated as the change in deviation per millimetre of surgical dose, and associations between baseline characteristics and both surgical success and dose response. 

Statistical analyses were performed using Python software. Continuous variables were summarised as means with standard deviations, and categorical variables as frequencies and percentages. Group comparisons used Mann-Whitney U tests for continuous variables. Predictors of surgical success were assessed using multivariate logistic regression, while predictors of surgical dose response were evaluated using multivariate linear regression. A p-value <0.05 was considered statistically significant. The analysis was conducted using complete-case analysis. Observations with missing values for variables required in a given analysis were excluded from that analysis. No imputation was performed, given the retrospective design and the relatively small sample size, in order to minimise the risk of overfitting. Continuous variables included in regression analyses were assessed for approximate linear relationships with outcomes through visual inspection of scatter plots and fitted regression lines. No major deviations from linearity were observed. Variables representing overlapping surgical measures (e.g., total surgical dose and surgical dose response) were not included simultaneously within the same regression model. This approach was used to minimise multicollinearity. As such, formal variance inflation factor (VIF) testing was not required.

## Results

Demographics and baseline characteristics

A total of 70 patients were included in this study. Demographics and preoperative clinical characteristics are listed in Table 1. The mean age at surgery was 8.0 ± 3.9 years. The mean follow-up duration was 12.1 ± 14.4 months. There were 40 females and 30 males. Visual acuity (VA) was normal (logMAR≤0.3) in 100% of recorded better eyes. In the worse or affected eyes, 85.1% had normal VA. The mean preoperative deviation was 37.69 ± 13.36 PD. The most common procedure was bilateral MR recession (bMR; 54/70, 81.4%). This was followed by unilateral MR recession and LR resection (R&R, 10/70, 14.3%), then unilateral MR recession (uMR, 3/70, 4.3%), unilateral LR resection (uLR, 2/70, 2.9%), and bilateral MR recession and unilateral LR resection (bMR & uLR, three-muscle surgery, 1/70, 1.4%). The most common type of esotropia (ET) was accommodative (38/70, 54.3%). Other types included infantile (20/70, 28.6%), acute acquired comitant ET (AACE) (4/70, 5.7%), consecutive (3/70, 4.3%), sensory (3/70, 4.3%), and cyclic (2/70, 2.9%).

**Table 1:** Demographic and preoperative clinical characteristics logMAR: logarithm of the minimum angle of resolution; PD: prism diopters; bMR: Bilateral MR Recession; R&R: Unilateral MR Recession and LR Resection; uMR: Unilateral MR Recession; uLR: Unilateral LR Resection; bMR & uLR: Bilateral MR Recession and Unilateral LR Resection; ET: Esotropia; AACE: Acute Acquired Comitant Esotropia *Visual acuity was documented in 67/70 patients

	n, (%)
Age at surgery (years), mean ± SD (range)	8.0 ± 3.9 (2- 17)
Total duration of follow-up (months)	12.1 ± 14.4 (1- 60)
Sex	
Female	40 (57.1)
Male	30 (42.9)
Visual acuity, logMAR, (better eye) *	
Normal ≤ 0.3	67 (100)
Mild > 0.3 - < 0.5	0 (0)
Moderate ≥ 0.5 - 1.0	0 (0)
Severe > 1.0 - 1.3	0 (0)
Blindness > 1.3	0 (0)
Visual acuity, logMAR, (worse eye)	
Normal ≤ 0.3	57 (85.1)
Mild > 0.3 - < 0.5	4 (6.0)
Moderate ≥ 0.5 - 1.0	3 (4.5)
Severe > 1.0 - 1.3	1 (1.5)
Blindness > 1.3	2 (3.0)
Preoperative deviation distance (PD), mean ± SD	37.69 ± 13.36
Type of surgery	
1. bMR	54 (81.4)
2. R&R	10 (14.3)
3. uMR	3 (4.3)
4. uLR	2 (2.9)
5. bMR & uLR	1 (1.4)
Type of ET	
1. Accommodative	38 (54.3)
2. Infantile	20 (28.6)
3. AACE	4 (5.7)
4. Consecutive	3 (4.3)
5. Sensory	3 (4.3)
6. Cyclic	2 (2.9)

Primary outcome: surgical success at six weeks

Overall, 35 patients (50%) achieved postoperative deviation ≤ 10 PD (Table 2). Overcorrection occurred in 1/70 (1.4%) of the cohort. In an unadjusted univariate analysis, a smaller preoperative deviation predicted surgical success (p = 0.012); however, this was not significant in the multivariate model. The multivariate logistic model included age, sex, preoperative deviation, type of surgery, and ET subtype. In the multivariate model, older age at surgery is associated with greater surgical success (p = 0.014, OR 1.37, 95% CI 1.06-1.76). Other variables (sex, preoperative deviation, type of surgery, and ET subtype) were not associated with surgical success. VA was excluded because there was no variation, and it could not be included in the multivariate model. Success rates vary across surgery types, ranging from 33% to 100%. Higher rates were observed in R&R and uLR procedures, but numbers were small and not significant. However, these differences did not reach statistical significance, likely due to small sample sizes in some groups.

**Table 2:** Postoperative outcomes ^a^Mann-Whitney U test. *Significant p-value

Study parameter	Success (N=35), n (%)	Failure (N=35), n (%)	p-value	Effect estimates (95% CI)
Mean preoperative deviation (PD) ± SD	34.09 ± 12.86	41.29 ± 13.04	0.012^a^*	-7.20 (-13.38 to -1.02)
Mean surgical dose response (PD/mm) ± SD	2.97 ± 2.25	1.81 ± 1.30	< 0.001^a^*	1.17 (0.28 to 2.05)
Total surgical dose (mm)	10.83 ± 2.80	11.14 ± 2.19	0.547^a^	-0.31 (-1.51 to 0.89)

The surgical dose response

**Figure 1:**
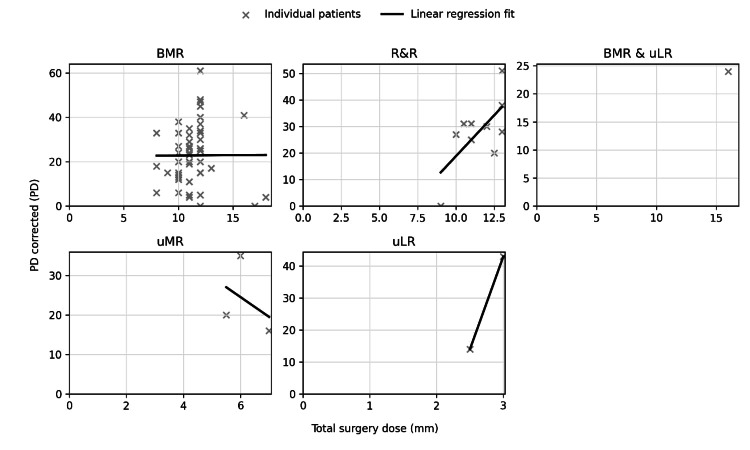
Dose-response curve by type of surgery. Univariate regression analysis of total surgical dose (mm) and distance correction (PD). Each point represents one patient. Dashed lines show the fitted linear regression for each surgery type

The mean surgical dose response was 2.39 ± 1.95 PD/mm. In the unadjusted univariate model, the mean dose response was higher in the success group than the failure group (2.97 PD/mm vs. 1.81 PD/mm, p < 0.001). In the multivariate linear regression, a larger preoperative deviation was associated with higher surgical dose-response (0.04 PD/mm per PD, p < 0.001, 95% CI 0.02-0.07) (Table 5). The surgical dose response was not statistically different among comparison types of surgery: bMR vs. R&R (Table 3). There was also no significant difference by ET subtype (accommodative ET vs. infantile ET, accommodative ET vs. AACE). Other types of surgery (uMR, uLR, and bMR & uLR) and ET subtypes (consecutive, sensory, and cyclic) were excluded in the multivariate model due to sparse data. Table 4 shows the multivariate analysis of the successful outcome. Figure 1 shows the dose-response curve by type of surgery.

**Table 3:** Outcome by type of surgery ^a^Surgery type shown for descriptive purpose only PD: prism diopters; bMR: Bilateral MR Recession; R&R: Unilateral MR Recession and LR Resection; uMR: Unilateral MR Recession; uLR: Unilateral LR Resection; bMR & uLR: Bilateral MR Recession and Unilateral LR Resection

Study parameter^a^	Success (N=35 patients), n (%)	Failure (N=35 patients), n (%)	Mean preop deviation (PD) ± SD	Mean surgical dose response (PD/mm) ± SD
1. bMR	26 (48.1%)	28 (51.9%)	37.56 ± 14.25	2.04 ± 1.15
2. R&R	6 (60.0%)	4 (40.0%)	39.00 ± 7.75	2.38 ± 1.04
3. uMR	1 (33.3%)	2 (66.7%)	38.33 ± 17.56	3.92 ± 1.79
4. uLR	2 (100.0%)	0 (0.0%)	32.50 ± 17.68	9.97 ± 6.18
5. bMR & uLR	0 (0.0%)	1 (100.0%)	40.00 ± 0.00	1.50 ± 0.00

**Table 4:** Multivariate analysis of successful outcome ^a^bMR was used as reference in the subanalysis of type of surgery *Significant p-value OR odds ratio; CI confidence interval

Study parameter	p-value	OR	95% CI
Age at surgery (years)	0.014*	1.37	1.06–1.76
Sex	0.647	1.33	0.39–4.51
Preoperative deviation distance (PD)	0.784	0.99	0.94–1.05
Type of surgery			
1. bMR^a^	Reference		
2. R&R	0.334	3.11	0.31–31.04
Type of ET			
1. Accommodative	Reference		
2. Infantile	0.987	1.01	0.20–5.08
3. AACE	0.797	1.42	0.10–20.64

**Table 5:** Multivariate analysis of surgical dose response ^a^bMR was used as a reference in the subanalysis of the type of surgery *Significant p-value CI confidence interval PD: prism diopters; bMR: Bilateral MR Recession; R&R: Unilateral MR Recession and LR Resection; ET: esotropia; AACE: Acute Acquired Comitant Esotropia

Study parameter	p-value	B (Coefficient)	95% CI
Age at surgery (years)	0.349	0.04	-0.05–0.14
Sex	0.560	-0.16	-0.70–0.39
Preoperative deviation distance (PD)	< 0.001*	0.04	0.02–0.07
Type of surgery			
1. bMR^a^	Reference		
2. R&R	0.423	0.35	-0.52–1.21
Type of ET			
1. Accommodative	Reference		
2. Infantile	0.666	0.14	-0.52–0.80
3. AACE	0.206	0.73	-0.42–1.88

## Discussion

In this single-centre cohort of 70 patients with horizontal esotropia (ET), 50% achieved postoperative alignment within ≤10 PD at six weeks, with an overcorrection rate of 1.4%. In the multivariate model, older age was a significant predictor of surgical success. However, the same trend was not observed in sex, VA, type of surgery, and ET subtype. Notably, the surgical dose-response was higher in the success group compared to the failure group, and a larger preoperative deviation was associated with a higher surgical dose-response. Furthermore, our cohort was predominantly female, and most had normal vision in both the better and worse/affected eyes. Finally, most patients had preserved VA, indicating generally good visual potential.

The overall early surgical success in our mixed ET cohort of 50% was lower than reported in other studies [2-5]. Homogeneous cohorts in infantile ET, partially accommodative ET (PAET), acquired non-accommodative ET, and acute acquired comitant ET (AACE) reported success rates ranging from 50% to 90%. Several differences between our study and these cohorts likely explain this discrepancy. First, most published series are homogeneous, focusing on a single diagnosis, such as infantile ET, PAET, or acquired ET, whereas our cohort deliberately included a mix of six ET subtypes [2-5]. Our cohort reflects the complexity and heterogeneity of a real-world tertiary strabismus practice. The pathophysiological differences in ET subtypes and differences in binocular potential respond differently to surgical dosage [10]. When combined into a single mixed group, the favourable outcomes seen in certain subtypes may dilute overall success rates. Second, many previous studies assess outcomes at 6-12 months or at final follow-up, whereas we focused on short-term surgical success at six weeks, which may underestimate the proportion of patients who stabilise within the success range [4,11]. Third, certain cohorts used an augmented surgical dosing in larger preoperative deviations or high accommodative convergence/accommodation ratio, whereas most of our patients underwent a standard approach regardless of preoperative deviation [7,12,13]. This was done to prevent overcorrection, and this is reflected by the low incidence of post-op overcorrection in our series. The low overcorrection rate reflects a safety-oriented surgical philosophy, which is important in paediatric populations. 

Our multivariate analysis identified that only older age at surgery was associated with greater surgical success. This is in keeping with observations in PAET, where older age and longer preoperative prism wear have been associated with higher surgical success [4,14]. This may be due to better compliance and better binocular potential. Additionally, sex, VA, preoperative deviation, and type of surgery were not associated with success, consistent with previous findings [3,4]. In contrast, a smaller preoperative deviation (< 50 PD) was associated with a successful surgical outcome in another study, likely due to a relatively easier surgical correction of smaller deviations [2]. Further, patients with a preoperative deviation <30 PD had lower reoperation rates and better alignment outcomes [15]. In our cohort, however, the mean preoperative deviation was already in the mid-range and relatively narrow (34.09 ± 12.86 PD in the success group vs. 41.29 ± 13.04 PD in the failure group), with few patients at very small or very large extremes. This restricted range may have limited our ability to detect a clinically meaningful effect of preoperative deviation on surgical success. 

Overall, the mean surgical dose response was 2.39 ± 1.95 PD/mm. This was lower than the cohorts that have higher rates of successful outcomes [4,6,7,16]. The reported dose-responses in the cohort study of AACE and infantile ET were 2.67 PD/mm and 3.91 PD/mm, respectively [6]. Infantile ET had a significantly higher surgical dose-response and responded more strongly per mm [6]. Similarly, in a cohort using augmented dosing in AACE, the dose-response was higher at 5.48 PD/mm in the augmented group of bMR compared to 4.39 PD/mm in the non-augmented group, with the association that an augmented surgical dose was associated with surgical success [7]. A higher surgical dose was associated with a successful outcome in the cohort of PAET assessing long-term outcomes of UMR [4]. In our cohort, the successful group exhibited a higher surgical dose-response than the failure group (2.97 ± 2.25 PD/mm vs. 1.81 ± 1.30 PD/mm), with an association between larger preoperative deviation and a greater dose-response. These findings suggest that a higher achieved dose-response is associated with success. 

Limitations

This study has several limitations. Its retrospective design introduces potential selection and information biases. The selection bias, lack of standardised surgical dosing, and standardised measurement may affect our surgical dose-modelling. Surgical allocation was not randomised but was based on surgeon preference and case complexity. Surgeries were performed by multiple surgeons and a few trainees; hence, we could not adjust for inter-surgeon effects. Surgeons have different measurement techniques, minor intraoperative adjustments, and thresholds for reoperation. This introduces systematic noise into the success rate and the surgical-dose response. Outcomes were assessed at six weeks. Our success rate may not reflect long-term surgical success, as studies have documented progressive drift after initial alignment. Hence, the success rate in our cohort may have been overstated and may decline with long-term follow-up. Sensory outcomes were incompletely documented, which precludes robust analysis of stereopsis. Data such as refractive error, AC/C ratio, and prism adaptation were also not documented, which limits the interpretability of the ET subtype. There could also be measurement variability in clinicians in measuring preoperative deviation and postoperative deviation, and referral bias in data collection. Given the relatively small cohort, including 35 successful cases, and the exploratory nature of the multivariable analyses, some sensitivity of model estimates to individual observations is possible. Models were therefore kept parsimonious, and results were interpreted cautiously as associations rather than causal inferences.

## Conclusions

Overall, this five-year retrospective study demonstrated a modest early alignment outcome in a real-world heterogeneous esotropia population. We demonstrated that 50% achieved alignment within ≤10 PD at six weeks and a low overcorrection rate (1.4%). Multivariate analysis found that older age at surgery was the only baseline characteristic associated with early surgical success, while other baseline variables, such as sex, VA, preoperative deviation, ET subtype, and type of surgery, were not predictive of surgical success. Surgical dose response appears clinically relevant, where successful cases showed a higher dose response, and larger preoperative deviations were independently associated with greater dose response, suggesting that correction achieved per millimetre of surgery is linked to early alignment outcomes. These findings highlight the difficulty of achieving consistent results in a heterogeneous tertiary population and support future prospective studies and longer follow-up to assess stability to optimise outcomes.
